# Supplementary material for: The interactome of intact mitochondria by cross-linking mass spectrometry provides evidence for coexisting respiratory supercomplexes
Source: Mol Cell Proteomics. 2017 Dec 8;17(2):216–32. doi: 10.1074/mcp.RA117.000470 (PMC5795388; doi:10.1074/mcp.RA117.000470)
Supplement: Supplemental Data [file supp_RA117.000470_133922_0_supp_23978_fzffwf.zip › spectra_annotation/mito_spectra_annotation/160-1-6-1-9-1.pdf]

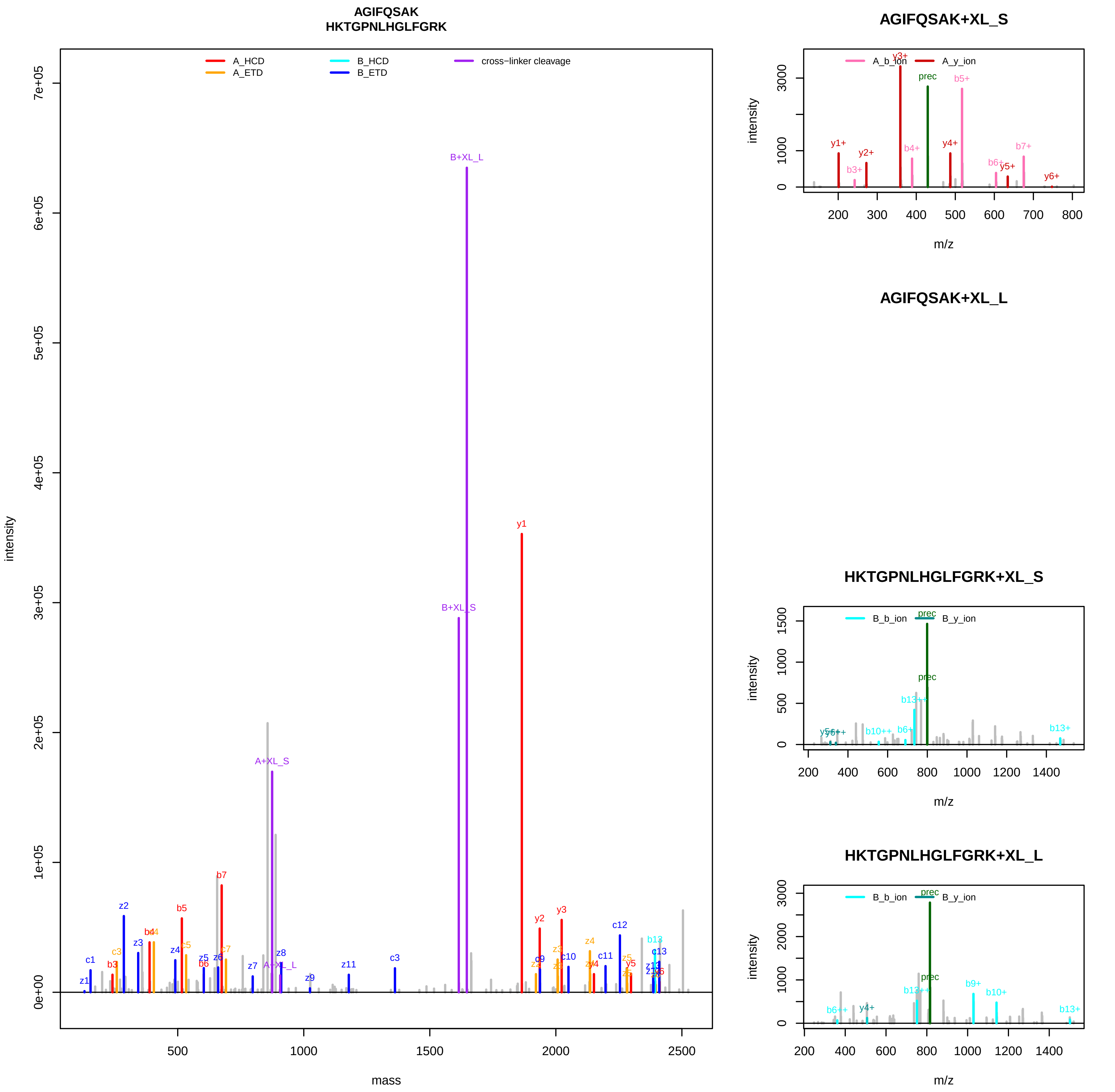

AGIFQSAK
HKTGPNLHGLFGRK
A_HCD
A_ETD
B_HCD
B_ETD
cross−linker cleavage
B+XL_L
B+XL_S
A+XL_S
A+XL_L
y1
y2
y3
y5
b3
b4
b5
b6
b7
c1
c3
c5
c7
c9
c10
c11
c12
c13
z1
z2
z3
z4
z5
z6
z7
z8
z9
z11
b13
intensity
mass
0e+00
1e+05
2e+05
3e+05
4e+05
5e+05
6e+05
7e+05
500
1000
1500
2000
2500
AGIFQSAK+XL_S
A_b_ion
A_y_ion
y1+
y2+
y3+
y4+
y5+
y6+
b3+
b4+
b5+
b6+
b7+
prec
intensity
m/z
0
1000
3000
200
300
400
500
600
700
800
AGIFQSAK+XL_L
HKTGPNLHGLFGRK+XL_S
B_b_ion
B_y_ion
prec
b13++
b10++
b6+
b13+
intensity
m/z
0
500
1000
1500
200
400
600
800
1000
1200
1400
HKTGPNLHGLFGRK+XL_L
B_b_ion
B_y_ion
prec
b6++
y4+
b13++
b9+
b10+
b13+
intensity
m/z
0
1000
2000
3000
200
400
600
800
1000
1200
1400
